# Effect of Au nano-particle aggregation on the deactivation of the AuCl_3_/AC catalyst for acetylene hydrochlorination

**DOI:** 10.1038/srep10553

**Published:** 2015-05-21

**Authors:** Bin Dai, Qinqin Wang, Feng Yu, Mingyuan Zhu

**Affiliations:** 1 School of Chemistry and Chemical Engineering of Shihezi University, Shihezi, Xinjiang 832000, P.R. China; 2 Key Laboratory for Green Processing of Chemical Engineering of Xinjiang Bingtuan, Shihezi, Xinjiang 832000, P.R. China

## Abstract

A detailed study of the valence state and distribution of the AuCl_3_/AC catalyst during the acetylene hydrochlorination deactivation process is described and discussed. Temperature-programmed reduction and X-ray photoelectron spectral analysis indicate that the active Au^3+^ reduction to metallic Au^0^ is one reason for the deactivation of AuCl_3_/AC catalyst. Transmission electron microscopy characterization demonstrated that the particle size of Au nano-particles increases with increasing reaction time. The results indicated that metallic Au^0^ exhibits considerable catalytic activity and that Au nano-particle aggregation may be another reason for the AuCl_3_/AC catalytic activity in acetylene hydrochlorination.

Acetylene hydrochlorination is an important process in the industrial production of vinyl chloride monomer, which is the primary raw material used to synthesize polyvinyl chloride, especially in China, because of the vast domestic coal resources and the increasing cost of petroleum^1^. HgCl_2_ supported on activated carbon is a common catalyst for industrial acetylene hydrochlorination. However, the HgCl_2_ catalyst is toxic and harmful to human health. Therefore, the exploitation of non-mercury catalysts to replace HgCl_2_ is of significant importance for the survival and development of the PVC industry through the acetylene route.

Numerous metal complexes, including Bi^3+^, Pt^6+^, Pt^4+^, and Pd^2+^, have recently been applied as candidate catalysts for acetylene hydrochlorination^2^,^3^,^4^,^5^. Hutchings^6^,^7^ reported that Au-based catalysts are the optimal metal chloride catalysts for acetylene hydrochlorination. However, the Au-based catalyst is easily deactivated over the course of the reaction.

Many investigations have attempted to elucidate the deactivation mechanism of AuCl_3_/AC catalyst in acetylene hydrochlorination. Hutchings proposed that the reduction of Au^3+^ to Au^0^ is the primary deactivation pathway under the operating condition of acetylene hydrochlorination^8^,^9^. Hutchings further disclosed the reason for active Au^3+^ reduction in acetylene hydrochlorination and proposed that acetylene was more easily adsorbed by AuCl_3_ catalyst compared with the hydrogen chloride species and that the strong adsorption of acetylene caused the reduction of Au^3+^ 10. Shen *et al.* reported similar results. These researchers determined that the initial coordination of hydrogen chloride with AuCl_3_ produced a calculated energy of -23.90 kJ mol^−1^, whereas acetylene in the vacant coordination site of AuCl_3_ generates a relative energy of -66.65 kJ mol^−1^. Therefore, the adsorption of acetylene on AuCl_3_ is relatively stronger than that of hydrogen chloride^11^. Zhang *et al.* suggested a detailed deactivation mechanism of AuCl_3_ catalyst for acetylene hydrochlorination. These researchers determined that the electron in the *p* orbital of acetylene transfers to the unoccupied molecular orbital of Au^3+^, thereby causing the reduction of Au^3+^ to its low-valence state via the loss of its Cl atoms^12^.

From the above-described literature, the reduction of active Au^3+^ ions to metallic Au^0^ is the primary reason for the AuCl_3_/AC catalyst deactivation in acetylene hydrochlorination. However, all of these investigators ignored an obvious fact. Au nano-particles are present in the deactivated catalyst. Thus, Au nano-particle aggregation may play a role in the AuCl_3_ catalyst deactivation because the particle size of Au nano-particles significantly affects catalytic performance, as described in many reports on heterogeneous catalysis^13^,^14^,^15^. In our previous work, we observed that the AuCl_3_ particle size after acetylene hydrochlorination is notably large^16^, and metallic Au^0^ (3-10) clusters may exhibit considerable catalytic activity based on the results of simulations using density functional theory^17^. Therefore, Au nano-particle agglomeration may also influence the catalytic performance of AuCl_3_ catalyst in acetylene hydrochlorination.

In this paper, we investigate the relationship between the metallic Au^0^ particle size and its catalytic performance to elucidate the deactivation mechanism of AuCl_3_/AC for acetylene hydrochlorination. A series of characterizations was performed to determine the valence state and distribution of the AuCl_3_/AC catalysts in various stages of catalyst life.

## Experimental Section

### Materials

Activated carbon (marked as AC, neutral, 40-60 mesh), HAuCl_4_·4H_2_O (with 47.8% Au content), C_2_H_2_ (gas, 98%), and HCl (gas, 99%) were used in this study.

### Catalyst preparation

The AuCl_3_/AC catalysts were prepared using an incipient wetness impregnation technique that used aqua regia as a solvent, as described in the literature^18^. The gold precursor, HAuCl·4H_2_O, was dissolved in aqua regia [3:1 HCl (Fisher, 32%): HNO_3_ (Fisher, 70%) by volume, 6.4 mL], and the solution was added dropwise with stirring to the activated carbon support (3.00 g) to obtain a catalyst with a final metal loading of 1 wt.%. The mixture was maintained for 24 h at room temperature, and then the product was dried for 18 h at 140 °C before being used as a catalyst.

The Au/AC catalyst (1 wt.% Au) was prepared using a colloidal deposition technique similar to that in the literature^19^, with a few modifications. First, 100 mL of distilled water was mixed with 3.2 mL of 2.43 × 10^−2^ M AuCl_4_^-^ solution, and then 5 mL of 0.1 M fresh NaBH_4_ solution was added dropwise. The solution was stirred for 2 h. Next, activated carbon (1.50 g) was added, and then the pH value was adjusted to 1.0 using 1.0 M HCl. Subsequently, the solution was vigorously stirred overnight. Finally, the mixture was washed with distilled water, and then dried in a vacuum oven at 80 °C to yield the Au/AC catalyst. Two samples were calcined for 4 h at 300 °C and at 800 °C (5 °C /min), the obtained samples were labeled as Au/AC-300 and Au/AC-800, respectively.

### Catalyst characterization

X-ray diffractometer data were collected using a Bruker D8 advanced X-ray diffractometer equipped with a Cu-K_α_ irradiation source (λ = 1.5406 Å) operating at 40 kV and 40 mA, with data collected over the 2*θ* scanning range between 20° and 90°. The morphologies of the samples were examined via transmission electron microscopy using a JEM 2010 electron microscope operating at an accelerating voltage of 200 kV, with a line resolution of 0.14 nm, and a point-to-point resolution of 0.23 nm. Temperature-programmed reduction was performed using a similar Micromeritic ASAP 2720 apparatus equipped with a TCD detector. The reducing gas was 10% H_2_ in Ar, with a flow rate of 40 mL min^−1^. The temperature range was from 50 °C to 400 °C, with a heating rate of 10 °C min^−1^. X-ray photoelectron spectrum data were recorded using an Axis Ultra spectrometer with a monochromatized Al-K_α_ X-ray source (225 W), a minimum energy resolution of 0.48 eV (Ag 3d_5/2_) and a minimum X-ray photoelectron spectrum analysis area of 15 μm.

### Catalytic performance evaluation

The catalytic performance during acetylene hydrochlorination was evaluated in a fixed-bed glass microreactor (i.d. of 10 mm) operating just above atmospheric pressure. A CKW 1100 temperature controller (Chaoyang Automation Instrument Factory, Beijing, China) regulated the reactor temperature. The reactor was purged with nitrogen to remove water and air in the reaction system before the reaction process. Hydrogen chloride gas was passed through the reactor at a flow rate of 20 mL min^−1^ to activate the catalyst. After the reactor was heated to 180 °C, acetylene (24.3 mL min^−1^) and hydrogen chloride (29.4 mL min^−1^) were fed through the heated reactor containing 2 mL of catalyst, with a gas hourly space velocity of 870 h^−1^. The reaction products were analyzed using a gas chromatograph (GC-2014C) produced by Shimadzu International Trading Company Ltd. (Shanghai). Analysis conditions: chromatographic column type of 2 m × Φ 4 mm, stuffing of GDX-301, column temperature of 120 °C, FID detector, detector and vaporizer temperature of 150 °C, and injection volume of 60 μL. The conversion of acetylene (**X**_**A**_) and the selectivity to vinyl chloride monomer (**S**_**VC**_) as the criteria of catalytic performance^20^ were defined as the equations (1) and (2), respectively.


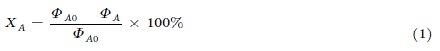



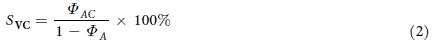


In the equations, 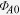 is defined as the volume fraction of acetylene in the raw gas, 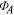 is defined as the volume fraction of remaining acetylene in the product gas, and 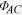 is the volume fraction of vinyl chloride in the product gas.

## Results and Discussion

**Figure 1:**
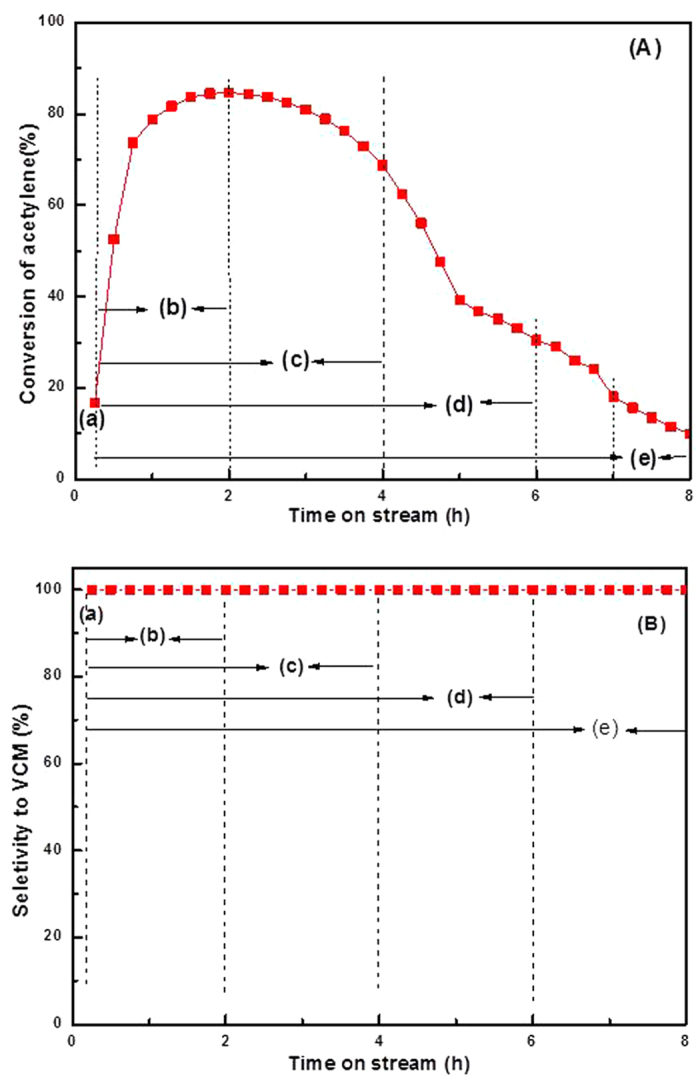
Acetylene conversion (A) and vinyl chloride monomer selectivity (B) during acetylene hydrochlorination catalyzed by (**a**) AuCl_3_/AC-0, (**b**) AuCl_3_/AC-2, (**c**) AuCl_3_/AC-4, (**d**) AuCl_3_/AC-6, and (**e**) AuCl_3_/AC-8. Reaction conditions: temperature = 180 °C, C_2_H_2_ gas hourly space velocity = 870 h^−1^, feed volume ratio V_HCl_/V_C2H2_ = 1/1.15.

**Figure 2:**
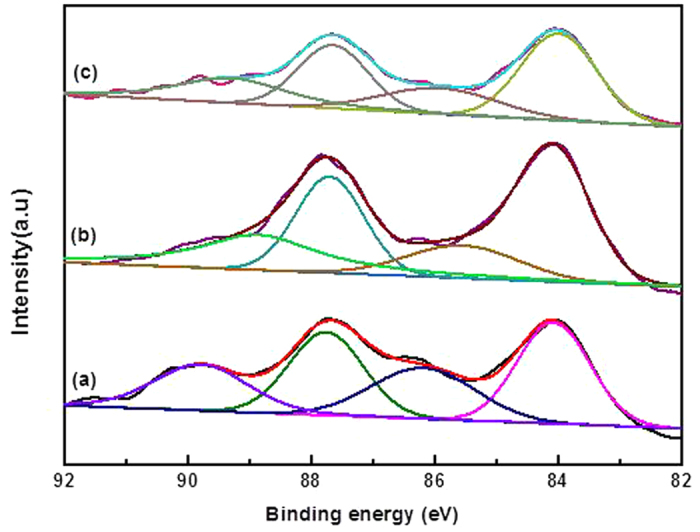
High-resolution X-ray photoelectron spectrum for Au 4f of (**a**) AuCl_3_/AC-0.5, (**b**) AuCl_3_/AC-1, and (**c**) AuCl_3_/AC-1.5.

To accelerate the deactivation process of Au-based catalysts, we applied both a gas hourly space velocity and a reaction temperature that were higher than those reported in the literature^21^. The catalysts were tested under fixed reaction conditions (C_2_H_2_/HCl = 1.15:1, gas hourly space velocity = 870 h^−1^, reaction temperature = 180 °C). The result is illustrated in Fig. 1A. The catalyst initially exhibited low conversion (ca. 16.71%), followed by an increase in activity after approximately 2 h, and then reached ca. 84.61% conversion. From the initial stage of the reaction (~ 2 h), there exists an activation period for the fresh AuCl_3_/AC catalyst during the acetylene hydrochlorination reaction. This result indicated that the most active sites for acetylene hydrochlorination are formed during this period. We characterized the AuCl_3_/AC catalyst during the initial stages of the reaction (~ 0.5 h, 1 h, and 1.5 h) by X-ray photoelectron spectrum experiments, and the results are listed in Fig. 2 and Table 1. From the results of Table 1, the compositions of Au^3+^ species in AuCl_3_/AC-0.5, AuCl_3_/AC-1 and AuCl_3_/AC-1.5 are observed to be 42.39%, 38.58% and 36.82%, respectively. Note that the content of the Au^3+^ species decreased, while the catalytic activity increased. This result indicated that Au^3+^ was not the only active site for acetylene hydrochlorination. The acetylene conversion decreased to ca. 68.70% after reacting for 4 h, and then the rate of acetylene conversion of the AuCl_3_/AC catalyst decreased to ca. 9.96% after 8 h, and the selectivity to vinyl chloride monomer of all AuCl_3_/AC catalysts was maintained at 99.99%, as shown in Fig. 1B. The results indicated that the AuCl_3_/AC catalyst was easily deactivated with increasing reaction time on the stream under these reaction conditions. Thus, we investigated the valence state and distribution of the catalyst at various stages of catalyst life. The catalyst was denoted as AuCl_3_/AC-x (where x represents the reaction time of 0, 2, 4, 6, and 8 h). The actual Au amount in AuCl_3_/AC-0, AuCl_3_/AC-2, AuCl_3_/AC-4, AuCl_3_/AC-6 and AuCl_3_/AC-8 catalysts was 1.09 wt.%, 0.98 wt.%, 1.02 wt.%, 1.04 wt.% and 1.01 wt.%, respectively, as determined by inductively coupled plasma-atomic emission spectrometry.

**Figure 3:**
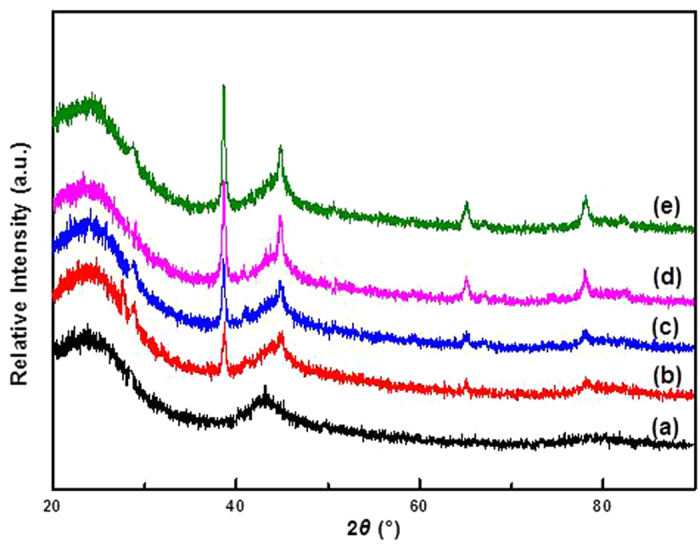
X-ray diffractometer patterns of (**a**) AuCl_3_/AC-0, (**b**) AuCl_3_/AC-2, (**c**) AuCl_3_/AC-4, (**d**) AuCl_3_/AC-6, and (**e**) AuCl_3_/AC-8.

X-ray diffractometer patterns of the representative AuCl_3_/AC-x catalysts are shown in Fig. 3. Two diffraction peaks in the pattern of AuCl_3_/AC-0 catalyst appear at 2*θ* = 24.4° and 43.7°, corresponding to the planes of (0 0 2) and (1 0 1) , respectively, of the AC support^22^. No obvious diffraction of Au^3+^ or metallic Au^0^ is observed in the fresh AuCl_3_/AC-0 catalyst, indicating that active Au^3+^ components are highly dispersed on the surface of the AC support. For AuCl_3_/AC-2, AuCl_3_/AC-4, AuCl_3_/AC-6, and AuCl_3_/AC-8 catalysts, obvious diffraction peaks appear at 38.5°, 44.7°, 64.8°, and 77.9° (2*θ*), respectively, corresponding to the planes (1 1 1), (2 0 0), (2 2 0), and (3 1 1), i.e., the diffraction peaks of metallic Au^0^ (JCPDS, No. 4-0784), for 38.5°, 44.7°, 64.8°, and 77.9° (2*θ*), respectively^23^. The X-ray diffractometer results show that Au^3+^ is reduced to metallic Au^0^ in acetylene hydrochlorination. Therefore, the reduction of an active Au^3+^ component is one reason for the deactivation of the AuCl_3_/AC catalyst in the acetylene hydrochlorination, which is consistent with the literature results^8^,^10^.

**Figure 4:**
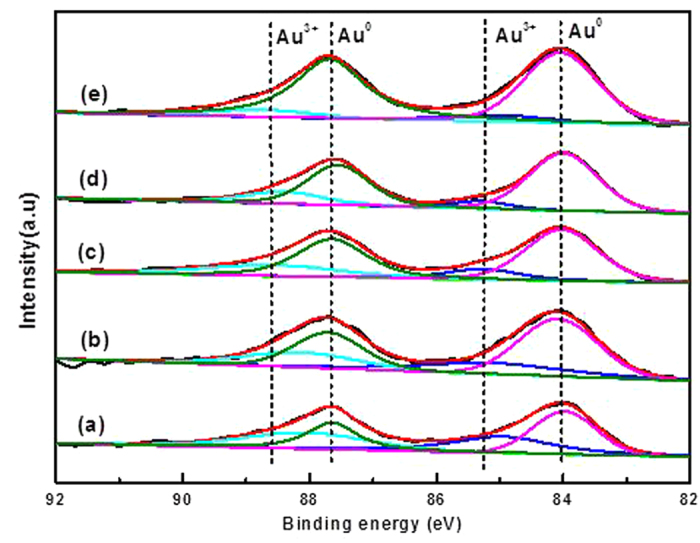
High-resolution X-ray photoelectron spectrum for Au 4f of (**a**) AuCl_3_/AC-0, (**b**) AuCl_3_/AC-2, (**c**) AuCl_3_/AC-4, (**d**) AuCl_3_/AC-6 and (**e**) AuCl_3_/AC-8.

In view of the above results, there still must be detailed investigations on the deactivation mechanism to make it clear whether a change of surface gold oxidation state was responsible for the deactivation for acetylene hydrochlorination. To correlate the bulk changes of the surface Au^3+^ in catalysts with the surface of the gold oxidation state, X-ray photoelectron spectroscopy was systematically performed for all AuCl_3_/AC-x catalysts. The deconvolution results in Fig. 4 indicate that each gold species shows two peaks, the gold species with the binding energy at 84.1 eV and 87.7 eV, due to Au 4f_7/2_, and the Au 4f_5/2_ spectrum of metallic Au^0^ 24; the spectral peaks at binding energies of 85.2 eV and 88.6 eV are assigned with Au^3+^. As shown in Fig. 4, the gold species on the surface of the catalysts exists as metallic Au and Au^3+^ states. The relative content of Au species in the fresh air and in the used catalysts, based on the deconvolution of X-ray photoelectron spectroscopy are tabulated in Table 2. As listed in Table 2, the amounts of gold species in the used catalysts differ greatly from those in the fresh one. Compared with the AuCl_3_/AC-0 catalyst, the amounts of Au^0^ increase from 50.55% to 65.47%, while the contents of Au^3+^ decrease after undergoing the reaction for 2 h. With increasing reaction time on the stream, the surface composition of approximately 89.38% of the Au^0^ species and 10.62% of the Au^3+^ species after 8 h, indicating that a great amount of Au^3+^ can be reduced to Au^0^ in the acetylene atmosphere. Therefore, the gold species on the catalysts surface exists as metallic Au^0^ and Au^3+^ states in acetylene hydrochlorination, while, approximately 54.65% of the Au^3+^ component is reduced to Au^0^ after reaction, which is one reason for the deactivation of AuCl_3_/AC catalyst in acetylene hydrochlorination.

**Figure 5:**
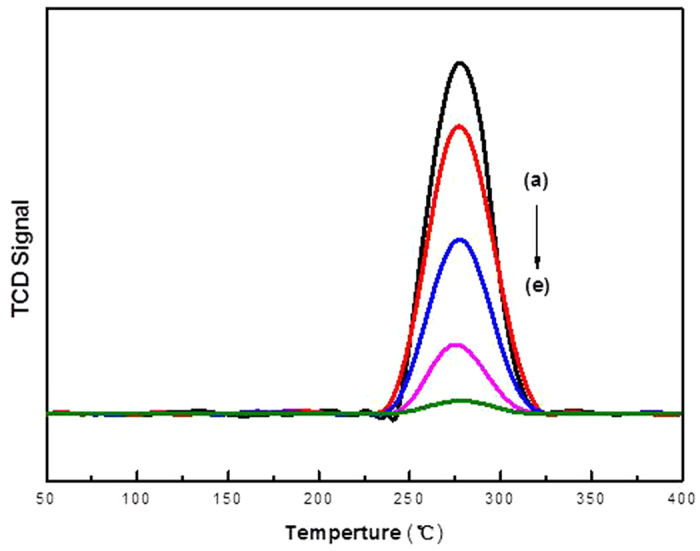
Temperature-programmed reduction profiles of (**a**) AuCl_3_/AC-0, (**b**) AuCl_3_/AC-2, (**c**) AuCl_3_/AC-4, (**d**) AuCl_3_/AC-6, and (**e**) AuCl_3_/AC-8.

**Figure 6:**
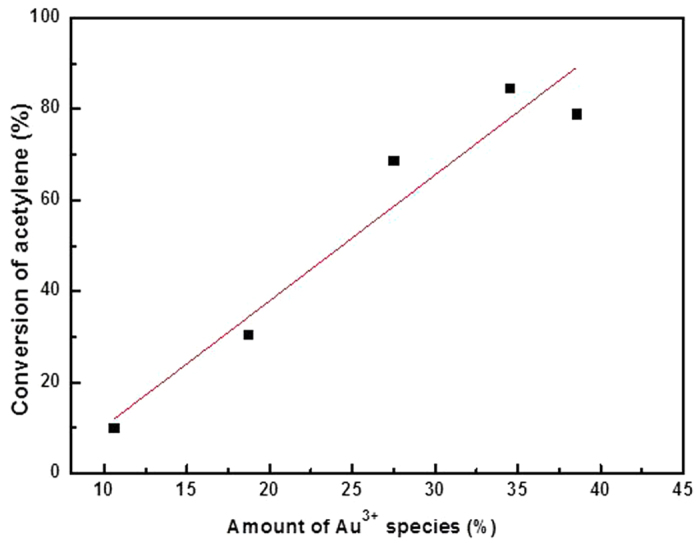
Correlation between the acetylene conversion and the amount of Au^3+^ species in the AuCl_3_/AC catalyst (Au^3+^ species were determined from Table 1 and Table 2).

To further quantify of the bulk Au^3+^ amount and the gold oxidation state during acetylene hydrochlorination reaction, temperature-programmed reduction was performed to monitor the amount of active Au^3+^ by integrating the reduction peak area of these AuCl_3_/AC-x catalysts at different reaction times during acetylene hydrochlorination. As shown in Fig. 5, all AuCl_3_/AC-x catalysts exhibit one characteristic reduction band between 250 °C and 350 °C with the peak centered at 277 °C, which is attributed to Au^3+^ reduction^25^. Determined from the reduction peak of temperature-programmed reduction, the Au^3+^ content was found to follow the same general trend obtained from X-ray photoelectron spectroscopy. Hence, the relative amounts of Au^3+^ decrease with increasing reaction time on stream, which is consistent with the order of the catalytic performance of the catalysts (shown in Fig. 1A). Moreover, the Au^3+^ content in these catalysts is well correlated with the acetylene conversion approximated by a single line, as shown in Fig. 6.

The above results indicate that approximately 13.59% of the Au^3+^ component is reduced to metallic Au^0^ when the reaction time is 2 h. If the Au^3+^ component is the only active site for acetylene hydrochlorination, and the acetylene conversion of AuCl_3_/AC-0 should be higher than that of the AuCl_3_/AC-2 catalyst. However, we observed a contradictory phenomenon in Fig. 1. The acetylene conversion of AuCl_3_/AC-2 (84.61%) is significantly higher than that of AuCl_3_/AC-0 (16.71%), as shown in Fig. 1. Therefore, we hypothesize that Au^3+^ is not the only active site for acetylene hydrochlorination, and metallic Au^0^ may also be active for this reaction.

**Figure 7:**
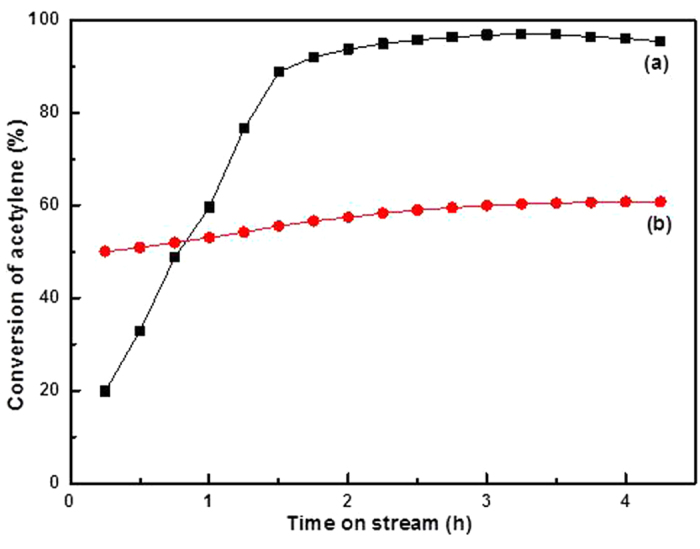
Acetylene conversion during acetylene hydrochlorination catalyzed by (**a**) AuCl_3_/AC and (**b**) Au/AC. Reaction conditions: temperature = 180 °C, C_2_H_2_ gas hourly space velocity = 870 h^−1^, and feed volume ratio V_HCl_/V_C2H2_ = 1.15.

To prove this hypothesis, we synthesized carbon support Au nano-particle with NaBH_4_ as a reducing agent and compared its catalytic performance for acetylene hydrochlorination with that of the AuCl_3_/AC catalyst. NaBH_4_ is a very strong reducing agent; thus, only metallic Au^0^ is present in the prepared Au/AC catalyst, as reported in the literature^26^. The catalytic performance of Au/AC catalyst for acetylene hydrochlorination was evaluated under a fixed reaction condition (C_2_H_2_/HCl = 1:1.15, gas hourly space velocity = 870 h^−1^, reaction temperature = 180 °C) compared with the AuCl_3_/AC catalyst. Notably, the acetylene conversion of AuCl_3_/AC is relatively higher than that in Fig. 1 because of the different reaction conditions. The acetylene conversion of the Au/AC catalyst is 57.1%, which is considerably lower than that of AuCl_3_/AC catalyst in Fig. 7. This result indicates that Au^3+^ is more active than metallic Au^0^, consistent with literature^25^,^27^. However, the catalytic activity of Au/AC is still higher than that of the AuCl_3_/AC-8 catalyst. If the reduction of Au^3+^ to Au^0^ is the only reason for the deactivation of AuCl_3_/AC catalyst, then the AuCl_3_/AC-8 catalytic activity should be close to that of Au/AC. Therefore, the possibility exists for another cause of the deactivation of AuCl_3_/AC catalyst for acetylene hydrochlorination, apart from the reduction of the active Au^3+^ component.

**Figure 8:**
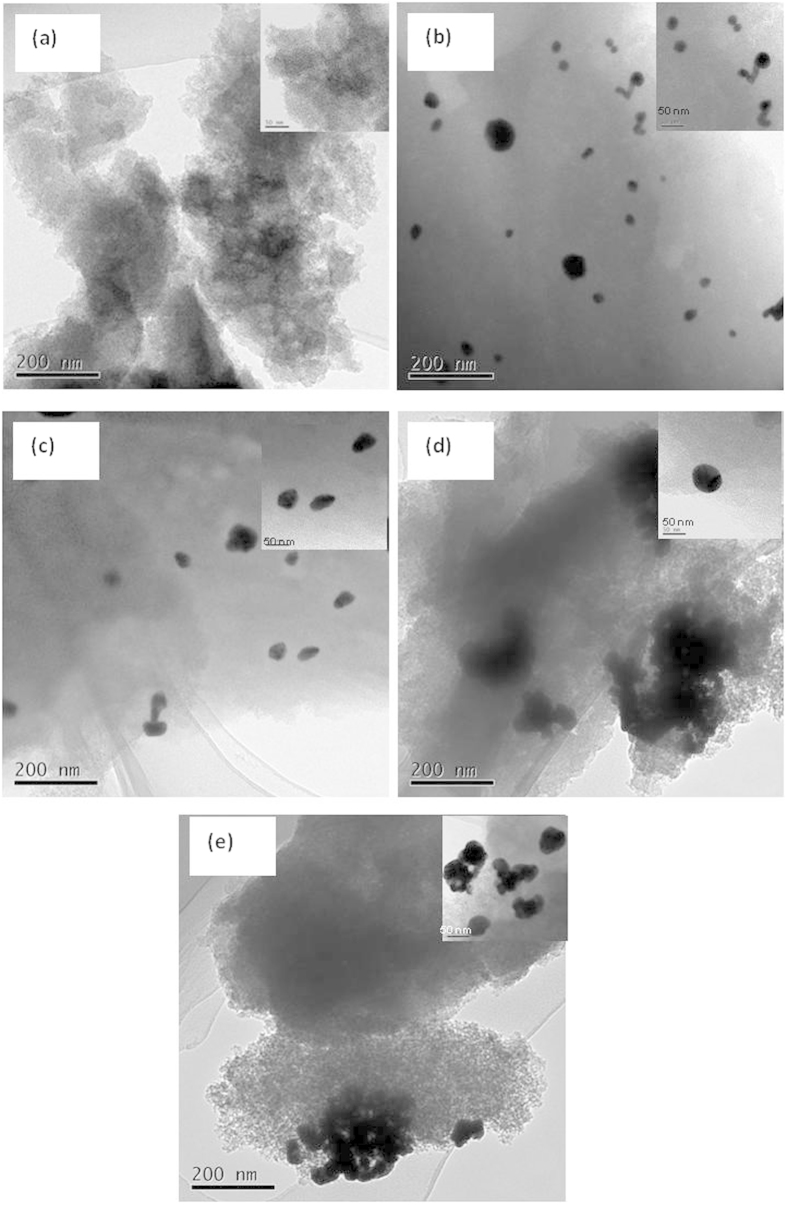
Transmission electron microscopy images of the catalysts: (**a**) AuCl_3_/AC-0, (**b**) AuCl_3_/AC-2, (**c**) AuCl_3_/AC-4, (**d**) AuCl_3_/AC-6, and (**e**) AuCl_3_/AC-8.

Representative transmission electron microscopy images of the AuCl_3_/AC-x catalysts are presented in Fig. 8 to determine the distribution of Au nano-particles in the acetylene hydrochlorination process. Fig. 8(a) shows no obvious presence of Au^0^ particles in the AuCl_3_/AC-0 catalyst, which demonstrates that the Au element is found in the form of Au^3+^ ion, which is invisible in the transmission electron microscopy micrographs. The X-ray diffractometer results confirm the transmission electron microscopy measurements and indicate that the AuCl_3_/AC-0 catalyst contains Au^3+^ only. While the X-ray photoelectron spectroscopy experiment exhibits an obvious Au^0^ peak in the sample, because the quantitative assessment of the gold oxidation state by X-ray photoelectron spectroscopy characterization alone is limited, the final state effects associated with particle size could significantly disturb the X-ray photoelectron spectroscopy features of Au^28^. For the AuCl_3_/AC-2, AuCl_3_/AC-4, and AuCl_3_/AC-6 catalysts, the average particle sizes of the Au^0^ species are 22.1, 34.2, 66.1 and 81.2 nm, respectively. The particle size of Au nano-particle increases with increasing reaction time on stream, which indicates that the Au nano-particles form aggregates during acetylene hydrochlorination. As previously discussed, metallic Au^0^ exhibits considerable catalytic activity for acetylene hydrochlorination, and the catalytic activity of the AuCl_3_/AC-x catalyst decreases with the reaction time increment during the acetylene hydrochlorination process. Therefore, Au nano-particle aggregation is another reason for the deactivation of AuCl_3_/AC catalyst in acetylene hydrochlorination.

**Figure 9:**
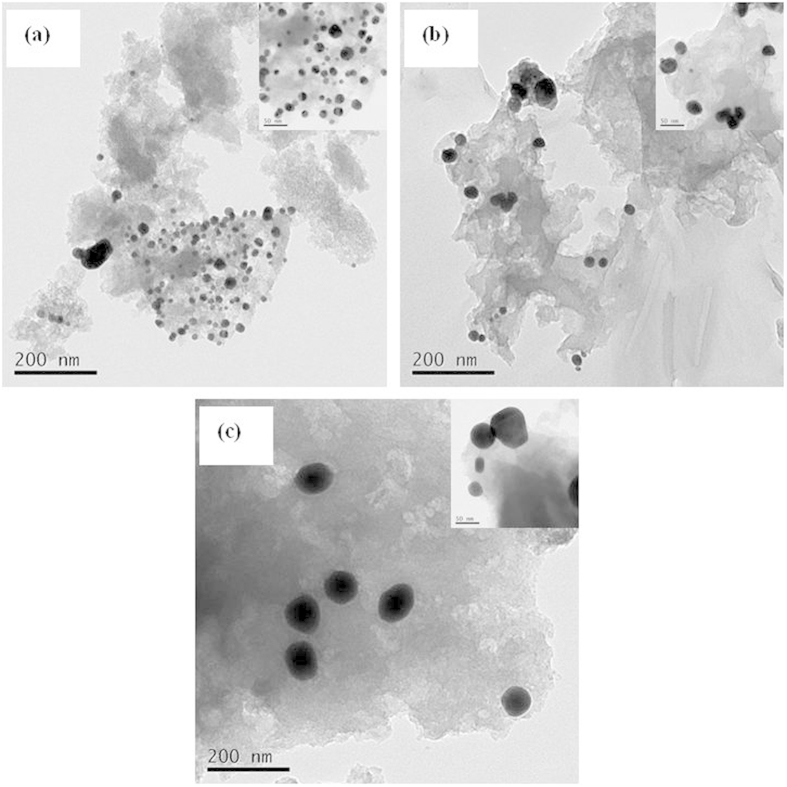
Transmission electron microscopy images of (**a**) Au/AC, (**b**) Au/AC-300, and (**c**) Au/AC-800.

**Figure 10:**
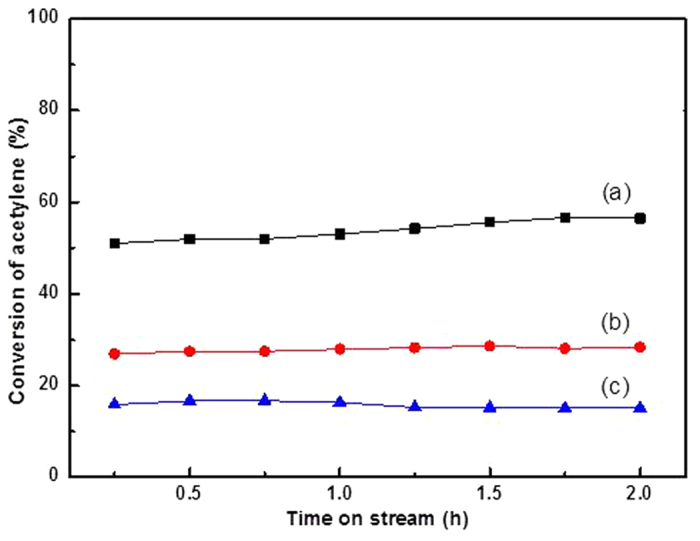
Acetylene conversion during acetylene hydrochlorination catalyzed by (**a**) Au/AC, (**b**) Au/AC-300, and (**c**) Au/AC-800. Reaction conditions: temperature = 180 °C, C_2_H_2_ gas hourly space velocity = 870 h^−1^, and feed volume ratio V_HCl_/V_C2H2_ = 1.15.

We prepared Au/AC catalysts with different particle sizes by subsequent heat-treatment under a nitrogen atmosphere at 300 °C and 800 °C for 4 h to support this viewpoint further. The particle sizes of the Au/AC, Au/AC-300, and Au/AC-800 catalysts are 21.3, 40.6, and 83.6 nm, respectively, as shown in Fig. 9. The catalytic activity of the Au catalysts decreases in the order of Au/AC > Au/AC-300 > Au/AC-800 (as shown in Fig. 10). The result determined that the Au/AC catalyst with larger particle size exhibited lower catalytic activity for acetylene hydrochlorination. This result also provides evidence that Au nano-particle aggregation is one of the reasons for the deactivation of the AuCl_3_/AC catalyst in acetylene hydrochlorination.

This paper reported the valence state and distribution of the AuCl_3_/AC catalyst at various stages of catalyst life for acetylene hydrochlorination. Metallic Au^0^ was found to exhibit considerable catalytic activity for acetylene hydrochlorination; as a result, Au nano-particle aggregation is another reason for the deactivation of AuCl_3_/AC catalyst, apart from the reduction of Au^3+^ component. Au nano-particle inhibition may be a development strategy for the exploration of a stable Au-based catalyst for acetylene hydrochlorination.

## Author Contributions

B.D. and M.Y.Z. designed experiments. F.Y. and Q.Q.W. prepared samples and carried out characterization and catalyst activity test. M.Y.Z. and Q.Q.W. contributed to the analysis and discussion for the results. M.Y.Z. and Q.Q.W. wrote the paper. All authors discussed the results and commented on the manuscript.

## Additional Information

**How to cite this article**: Dai, B. *et al.* Effect of Au nano-particle aggregation on the deactivation of the AuCl_3_/AC catalyst for acetylene hydrochlorination. *Sci. Rep.* **5**, 10553; doi: 10.1038/srep10553 (2015).

## Figures and Tables

**Table 1:** Composition of the Au species obtained by deconvolution of the Au 4f peaks.

**Catalysts**	**Composition (%)**	
	**Au**^**0**^	**Au**^**3+**^
AuCl_3_/AC-0.5	57.61	42.39
AuCl_3_/AC-1	61.42	38.58
AuCl_3_/AC-1.5	63.18	36.82

**Table 2:** Composition of the Au species obtained by deconvolution of the Au 4f peaks.

**Catalysts**	**Composition (%)**	
	**Au**^**0**^	**Au**^**3+**^
AuCl_3_/AC-0	50.55	49.45
AuCl_3_/AC-2	65.47	34.53
AuCl_3_/AC-4	72.48	27.52
AuCl_3_/AC-6	81.27	18.73
AuCl_3_/AC-8	89.38	10.62
